# Voxel-Wise Perfusion Assessment in Cerebral White Matter with PCASL at 3T; Is It Possible and How Long Does It Take?

**DOI:** 10.1371/journal.pone.0135596

**Published:** 2015-08-12

**Authors:** Mikjel Johannes Skurdal, Atle Bjørnerud, Matthias J. P. van Osch, Wibeke Nordhøy, Jim Lagopoulos, Inge Rasmus Groote

**Affiliations:** 1 Department of Neurology, Faculty Division, Akershus University Hospital, University of Oslo, Lørenskog, Norway; 2 The Intervention Center, Oslo University Hospital, Rikshospitalet, Oslo, Norway; 3 Department of Physics, University of Oslo, Oslo, Norway; 4 Department of Radiology, Leiden University Medical Center, Leiden, The Netherlands; 5 C.J. Gorter Center for High Field MRI, Leiden, The Netherlands; 6 Brain and Mind Research Institute, Sydney Medical School, Sydney, Australia; 7 Department of Psychology, Institute of Social Sciences, University of Oslo, Oslo, Norway; University of California, San Francisco, UNITED STATES

## Abstract

**Purpose:**

To establish whether reliable voxel-wise assessment of perfusion in cerebral white matter (WM) is possible using arterial spin labeling (ASL) at 3T in a cohort of healthy subjects.

**Materials and Methods:**

Pseudo-continuous ASL (PCASL) with background suppression (BS) optimized for WM measurements was performed at 3T in eight healthy male volunteers aged 25–41. Four different labeling schemes were evaluated by varying the labeling duration (LD) and post-labeling delay (PLD). Eight slices with voxel dimension 3.75x3.75x5 mm^3^ were acquired from the anterosuperior aspect of the brain, and 400 image/control pairs were collected for each run. Rigid head immobilization was applied using individually fitted thermoplastic masks. For each voxel in the resulting ASL time series, the time needed to reach a 95% significance level for the ASL signal to be higher than zero (paired t-test), was estimated.

**Results:**

The four protocols detected between 88% and 95% (after Bonferroni correction: 75% and 88%) of WM voxels at 95% significance level. In the most efficient sequence, 80% was reached after 5 min and 95% after 53 min (after Bonferroni correction 40% and 88% respectively). For all protocols, the fraction of significant WM voxels increased in an asymptotic fashion with increasing scan time. A small subgroup of voxels was shown to not benefit at all from prolonged measurement.

**Conclusion:**

Acquisition of a significant ASL signal from a majority of WM voxels is possible within clinically acceptable scan times, whereas full coverage needs prohibitively long scan times, as a result of the asymptotic trajectory.

## Introduction

Arterial spin labeling (ASL) is a rapidly developing magnetic resonance imaging (MRI) method used to measure and quantify cerebral blood flow (CBF) using magnetically labeled blood water as an endogenous tracer [1]. Unlike contrast agent-based techniques, this method allows repeated measurement of CBF in a non-invasive fashion, with adequate temporal and spatial resolution [2]. ASL has been successfully employed to quantify CBF in cerebral gray matter (GM) in healthy subjects as well as in numerous clinical cohorts (e.g. [3,4]) However, ASL measurements in white matter (WM) are less frequently reported (e.g. [5–7]), but there is an increasing interest in using ASL for studies of aberrant WM perfusion in ageing as well as disease states, such as dementia spectrum disorders, multiple sclerosis, hydrocephalus and ischemia.

Due to the low intrinsic signal difference between the control and labeled images, the voxel-wise assessment of WM CBF using ASL at 3T has proven to be challenging. This is partly because of the arterial transit times are significantly longer in WM than in GM [8] which leads to increased T_1_ relaxation before readout and consequently to a reduction of available WM ASL signal. In addition, the WM perfusion itself is 2–4 times smaller than in GM [9–11]. Single WM large-voxel acquisition and higher field strengths have been suggested to overcome these limitations [12,13].

Three bodies of work have specifically addressed the validity of measuring voxel-wise perfusion in WM using ASL at 3T. van Gelderen et al [8] used pulsed ASL and simulations to argue that proper voxel-wise assessment of CBF in WM is not possible due to the need for a high spatial resolution to avoid partial volume (PV) effects at the GM boundary, the low signal-to-noise (SNR) obtainable from WM compared to GM, the even lower SNR due to the high spatial resolution, as well as the prolonged transit times in WM affecting the label in a deleterious manner. With the introduction of pseudo-continuous (PC) labeling technique [14] combined with background suppression (BS) of static brain tissue [15], van Osch et al [9] demonstrated that a ten minute acquisition with a sequence optimized for GM provides reliable signal at 95% significance level in 70% of the WM voxels, whilst a power calculation indicated that a valid measurement of all voxels within the WM would be possible using a 20 minute long acquisition. Finally, Wu et al [16] optimized a high spatial resolution PCASL sequence for WM yielding a robust signal from a major part (~60%) of the WM voxels in seven minutes.

In addition to the PC labeling technique and BS, 3D image readout in ASL is a recent technique to further boost SNR, mainly by offering optimized utilization of BS [17]. 3D readout modules come at the expense of increased in-plane and through-plane image blurring leading to significant overestimation of WM CBF and underestimation of GM CBF. This makes it a less desirable alternative in terms of precise measurements, and further work needs to be done to radically sharpen the point-spread function (PSF) in 3D acquisitions schemes, especially given that suboptimal PSF has been shown to significantly corrupt WM voxel-wise perfusion assessments even when using 2D readout modules [13,18].

In the piloting phase of a dementia WM ASL study, the opportunity was taken to fully explore the feasibility of PCASL at 3T for the purposes of measuring voxel-wise CBF in WM. Discrepancies from literature (i.e. [9]) were listed, and an experimental assessment of actual necessary scan times was formalized. The study focuses on the feasibility of acquiring an acceptable voxel-wise raw ASL signal, while not specifically addressing additional problems such as PV effects, PSF and perfusion quantification issues, which are related but separate issues. This study is intended to be an experimental continuation of the study of van Osch et al [9].

## Material and Methods

### Subjects

The study was approved by the regional committees for medical and health research ethics south east (Norway), and written informed consent was obtained from the participants before study entry in compliance with the PLoS consent form. The individual in this manuscript has given written informed consent to publish these case details as outlined in PLoS consent form.

Eight healthy male volunteers (mean age = 33 years, range = 25–41 years) were recruited for the scanning procedures. All participants were experienced test subjects with no tendency of claustrophobia in the scanner environment so as to comply with the special head immobilization procedures employed and the prolonged scanning duration. None had general contraindications against undergoing MRI and none suffered from any neurological or psychiatric condition. Each participant underwent four separate experiments as described below.

### MRI protocols and scanning procedures

All scanning was performed on a research-dedicated 3T Philips Achieva (Philips Healthcare, Best, the Netherlands) MRI scanner using an 8-channel SENSE head coil (InVivo, Gainsville, Florida).

The imaging protocol consisted of a backbone sequence upon which variations in labeling duration (LD) and post-labeling delay (PLD) were made for the four different experiments as summarized in Table 1.

**Table 1:** Experiment characteristics.

Experiment	1	2	3	4
LD (ms)	1650	1650	2150	2150
PLD (ms)	1500	2000	1500	1000
TR (ms)	3500	4000	4000	3500
BS1 (ms)	1672	1671	2390	2261
BS2 (ms)	2803	3164	3366	2989
Scan time (min)	46.6	53.3	53.3	46.6

Abbreviations: LD = labeling duration, PLD = post labeling delay, TR = time repetition, BS = Background suppression pulse; time after start of acquisition

The backbone sequence was developed from a sequence described earlier [9] that has become the preferred sequence in the majority of recent clinical studies using PCASL on the Philips platform. The sequence was slightly modified with larger voxels and lower SENSE factor to theoretically increase SNR[19]. Total acquisition times, including localizers and additional scans, were approximately one hour for each experiment.

**Fig 1:**
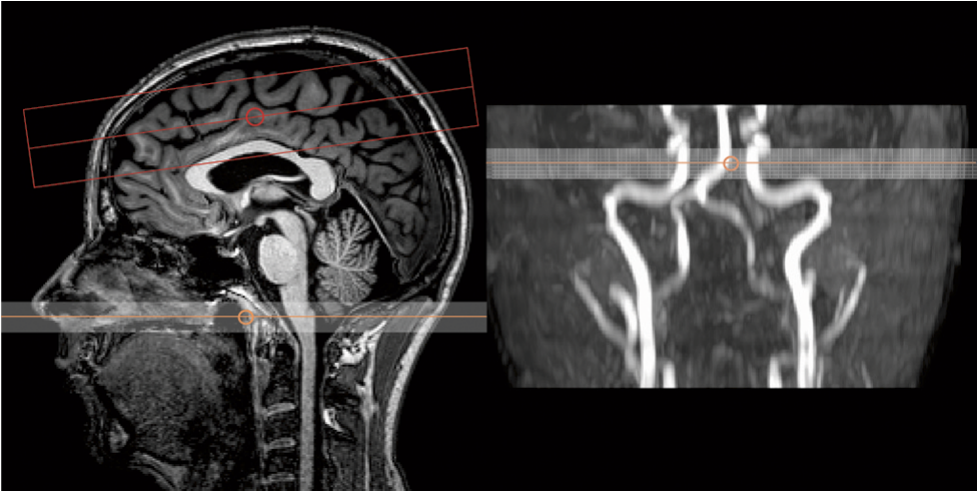
Imaging area and labeling position. Left image showing imaging area and labeling region, and right image demonstrates the placement of the label based on a quick time-of-flight angiogram.

The PCASL preparation consisted of labeling with a train of Hanning-shaped RF pulses (flip angle 18°, duration 0.5 ms) with an inter-pulse interval of 1 ms in a balanced gradient scheme [20]. The label was positioned orthogonally to the basilar and internal carotid arteries based on a 1.5-minute time-of-flight angiogram of the neck region (Fig 1).

BS was achieved by applying a saturation pulse before labeling and two inversion pulses embedded in the PLD period (Table 1). As the utilized image readout module was in 2D, this gave optimal BS in the first slice, and progressively incomplete suppression in ascending slices due to T_1_ relaxation. For this reason, only eight slices were acquired (rather than full brain) in order to have reasonable BS in all acquired data and decreases the signal fluctuation level by limiting the background signal.

Single-shot gradient echo echo-planar-imaging (EPI) with parallel imaging was used for image readout. Eight contiguous 5 mm slices with an in-plane resolution of 3.75x3.75 mm^2^ and a SENSE factor of 2 were acquired from the anterosuperior aspects of the brain (Fig 1), where the largest regions of WM in the brain are located, offering the least amount of PV signal corruption. The minimum achievable echo time of 10.9 ms was used, and the repetition time and total scan time varied for each experiment and are summarized in Table 1. The number of signal averages (NSA) was 400 label/control pairs for the four ASL experiments.

**Fig 2:**
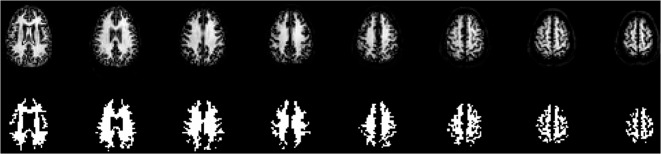
WM identification: Upper row showing SIR images from a representative subject, lower row shows the segmented WM.

For identification of WM, a separate image set using identical EPI acquisition parameters as the backbone sequence was acquired, prepared with a single inversion recovery (SIR) preparation pulse 1800 ms before readout, to yield an image set exactly registered to the ASL time series for WM segmentation purposes. The SIR sequence effectively suppressed GM, leaving very strong signal in WM and an intermediate signal in voxels of mixed tissue origin, and was used to segment voxels of interest and generate WM masks in perfect register with a minimum of PV contaminated voxels (Fig 2). This approach was chosen in preference to the more conventional method of using WM masks segmented from high-resolution T_1_ images, as gradient echo EPI images are invariably distorted and co-register poorly to T_1_ images, giving potentially corrupted WM selection.

**Fig 3:**
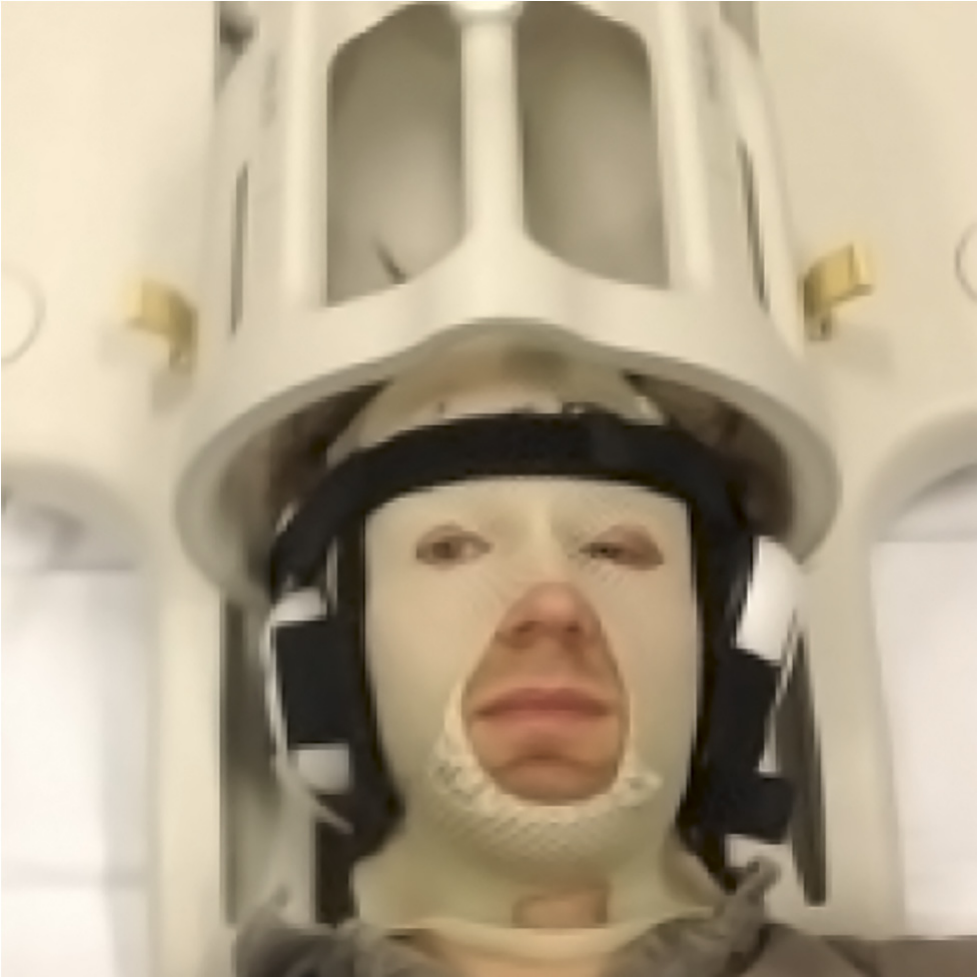
Head immobilization set-up, with a subject strapped to the coil using individual thermoplastic mold and three Velcro attachments.

Head immobilization for the prolonged scans was achieved using individually molded thermoplastic face masks (Orfit Industries, Wijnegem, Belgium) with openings for the mouth and eyes, strapped to the head coil with a stiff Velcro band over the forehead and two additional elastic Velcro bands to secure the head to the coil bucket as shown in Fig 3. Headphone hearing protection was embedded in the thermoplastic masks and used together with earplugs. This provided exceptional head immobilization to keep intra-session head movement at minimum levels for the prolonged scan sessions. The total PCASL-scan time for each subject was (2x46 + 2x53) = 198 minutes for the four experiments.

### Analysis

Image analysis was done using the nordicICE software tool (NordicNeuroLab AS, Norway) with in-house developed plugins for ASL- and statistical analysis, except for the motion correction, which was done using an algorithm from the FSL software tool collection [21]. All images where separated in label and control series before motion correction with MCFLIRT [22], and thereafter voxel-wise label/control subtraction was performed to create a time series of ASL images. The WM voxels exhibiting significant signal in the ASL images were identified using a one-tailed paired t-test with α = 0.05. A one-tailed test was used since the objective was to identify voxels exhibiting an ASL-effect, i.e. where the signal from the control images was significantly greater than the signal from the labeled images (positive t-values). A significance level (α) of 0.05, not correcting for multiple comparisons, was used following the argument proposed previously [9] that the null hypothesis for the test is that the label-control signal difference for each voxel under investigation is not significantly different from zero, irrespective of the other WM voxels. In order to test the influence of different significance levels, the analysis for experiment 3 was repeated with α = 0.01 and also using Bonferroni correction, thereby covering the entire range of realistic p-value cutoffs.

In order to investigate in more detail the effect of increasing NSA on the distribution of WM ASL signal, histogram analysis of the distribution of t-values in the entire WM mask as a function of NSA was performed. The area under the histogram distributions from the individual subjects was normalized to one and the range of t-values was fixed to -10 to +50 (based on initial investigation of the range of t-values present in the data) in steps of 10 (60 bins). A mean population t-value histogram was then obtained by averaging the values across all subjects for each normalized bin.

Based on the shape of the plots of percent significant WM voxels (y) as a function of NSA (x), this plot was fitted to different model functions reflecting the observed asymptotic approach to a maximum value. The following function types were tested: 1) exponential recovery:

$$y=a\cdot \left(1-e^{-bx}\right)$$ (1)

2) logarithmic dependency:

y=a⋅logx (2)

and 3) a fractional recovery function:

$$y=\frac{ax}{b+x}$$ (3)

where a and b are constants.

The function found to give the best fit was then chosen to describe the relationship. Correlation between NSA and average WM t-value was fitted to a function of the form y = K√NSA where K is a constant. The goodness-of-fit for both fits was assessed in terms of the residual error of the regression as well as confidence intervals for the fits. Curve fitting was performed in MiniTab 17 (Minitab Inc. USA).

**Fig 4:**
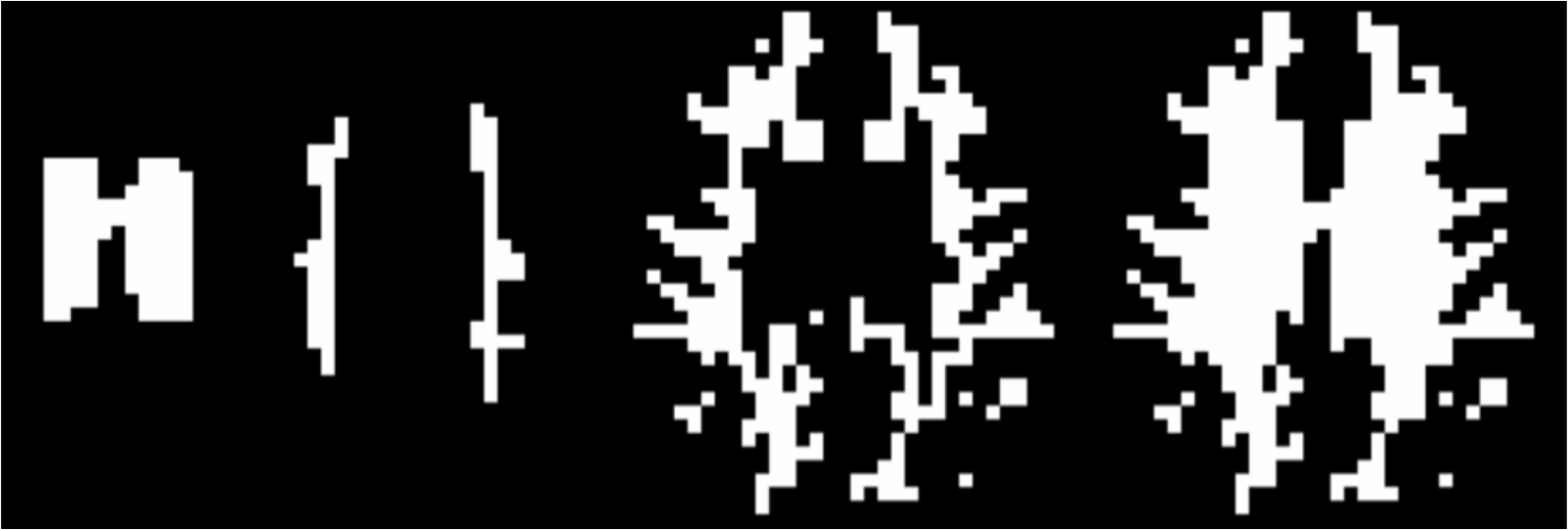
Sub-masks from left to right: Periventricular WM, deep WM, peripheral WM and entire WM.

A proper group registration approach was impossible due to the partial brain acquisition approach. To elaborate on the filling pattern of the WM voxels, each mask was divided into three sub-masks. The first sub-mask was defined by identifying the ventricles in the SIR image and expanding one voxel outwards in all directions, yielding a periventricular mask. The second sub-mask consisted of all peripheral voxels in the full WM mask, i.e. all WM voxels directly bordering GM. The third sub-mask was all remaining WM voxels (“deep” WM). Sub-masks from one representative subject are shown in Fig 4. The same t-test as described above was performed on each sub-mask.

## Results

All scans were technically successful, and with minimum head movement as reported by MCFLIRT: relative movement (mm): 0.08 standard deviation (s.d.) 0.02, median 0.07; absolute movement (mm) 0.25 s.d. 0.10, median 0.23.

**Fig 5:**
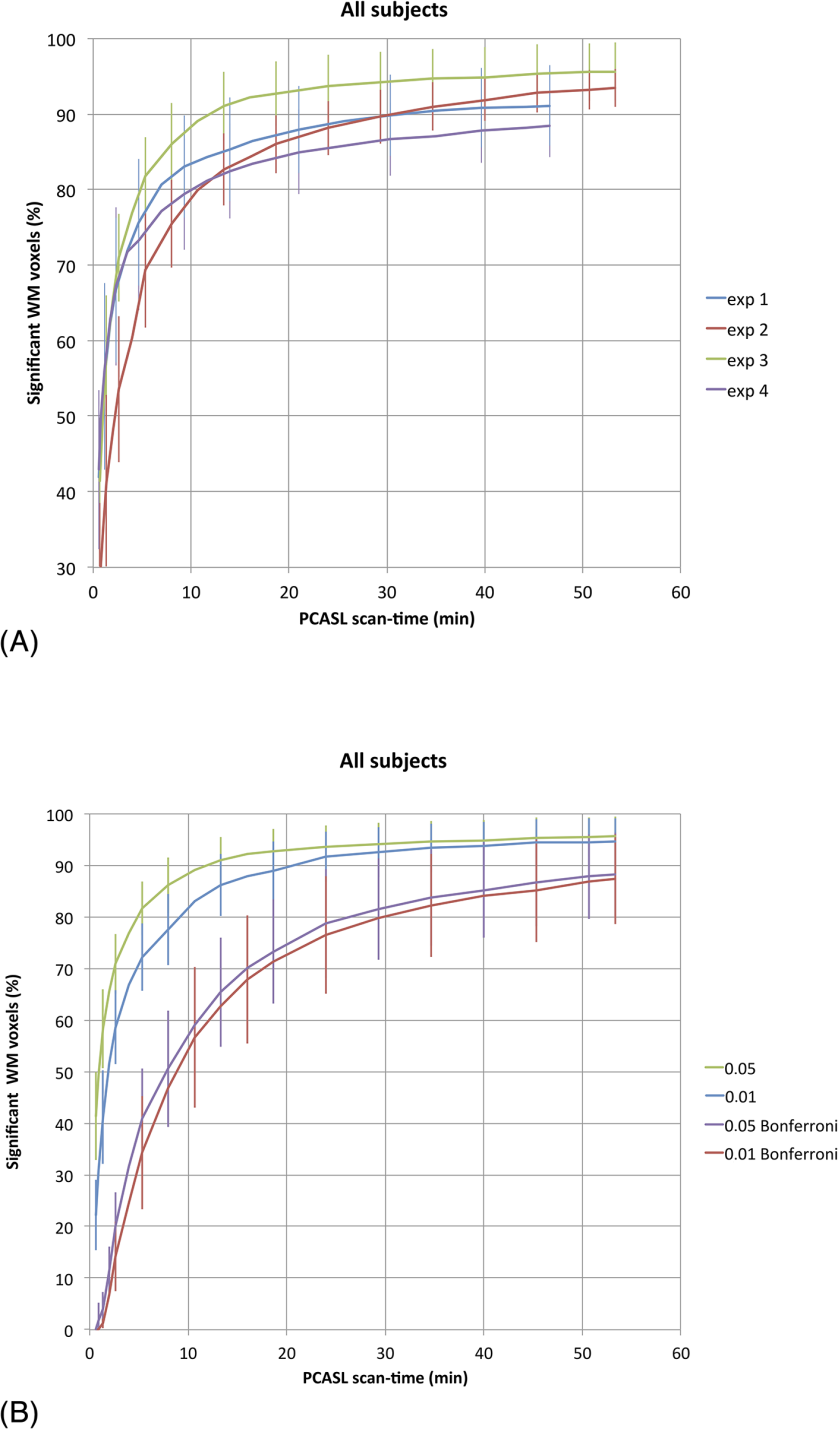
A) Average number of voxels exhibiting ASL signal larger than zero at a 95% significance level as a function of scan time in minutes for the four experiments. B) The effect of different statistical threshold levels on the results of experiment 3. Green line = t-value 0.05, uncorrected, blue line = t-value 0.01, uncorrected, purple line = t-value 0.05, Bonferroni-corrected, red line = t-value 0.01, Bonferroni-corrected.

The average percentage of voxels with statistically significant signal larger than zero at a 95%-level as a function of scan-time for the four sequences is shown in Fig 5A. The same plot at 99% significance level, as well as with Bonferroni-corrections is shown in Fig 5B for one selected sequence (experiment 3). The presented data are the averaged findings with s.d. over the eight subjects.

**Fig 6:**
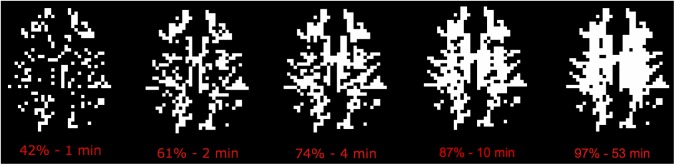
WM voxels different from zero at 95% significance level as a function of time in a representative subject for experiment 3.

For all the experiments there was an initial steep increase in number of WM voxels exhibiting significant perfusion signal, and experiment 3 (LD/PLD = 2150/1500) gave both the most rapid initial increase and highest total percentage of significant voxels, where 80% was reached after 5 min and 95% after 53 min (40% and 88% after Bonferroni correction, respectively) as illustrated in Fig 6.

**Fig 7:**
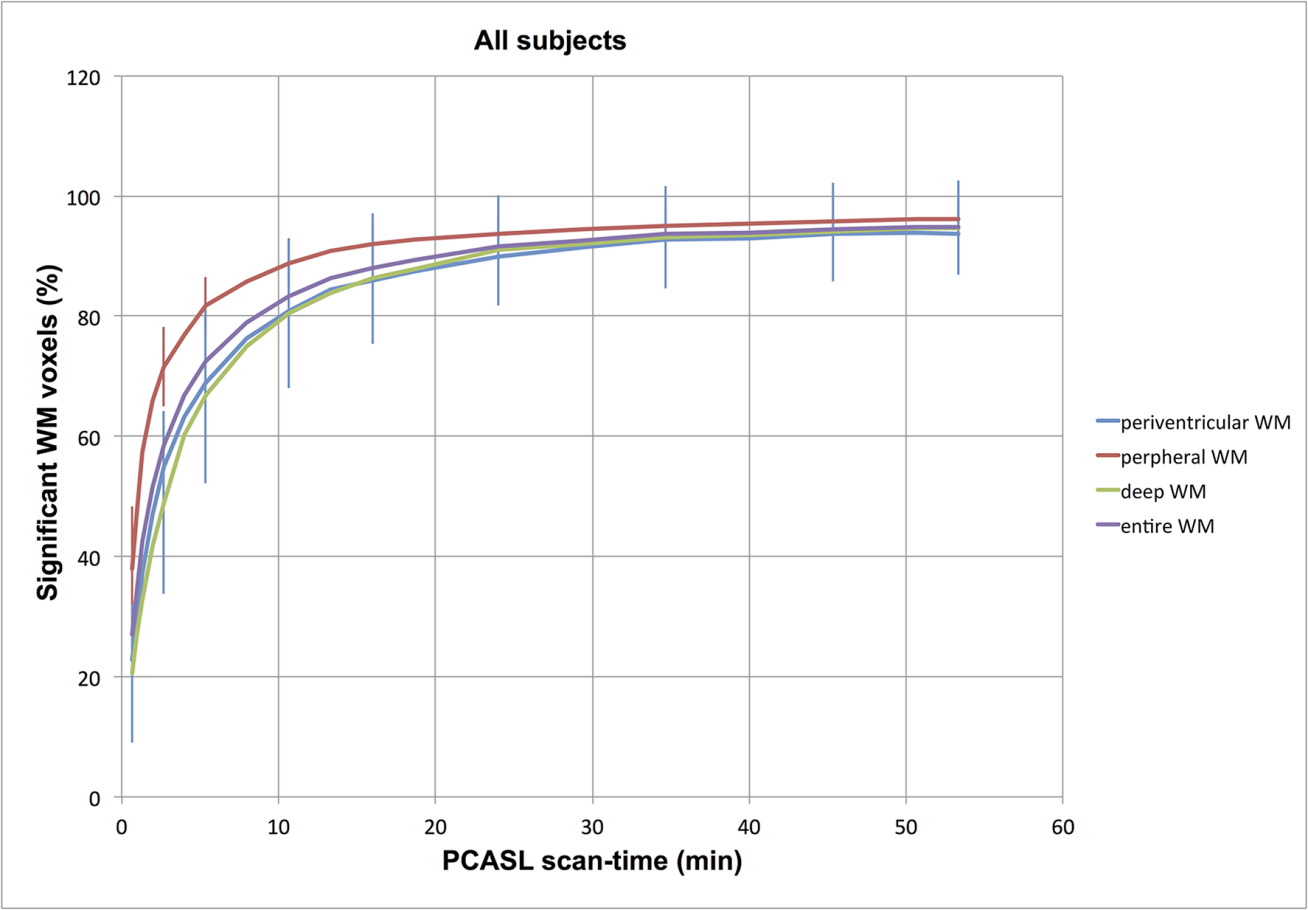
Differential filling of the three sub-masks in Experiment 3, suggesting that superficial WM has a quicker filling progression than deeper aspects of WM. Red line = peripheral WM mask, blue line = periventricular WM mask, green line = “deep” WM mask, purple line = entire WM.

The analysis of the filling pattern of the voxels in the three sub-masks, indicated that deep WM has a vaguely slower filling progression than peripheral voxels, and is illustrated in Fig 7.

**Fig 8:**
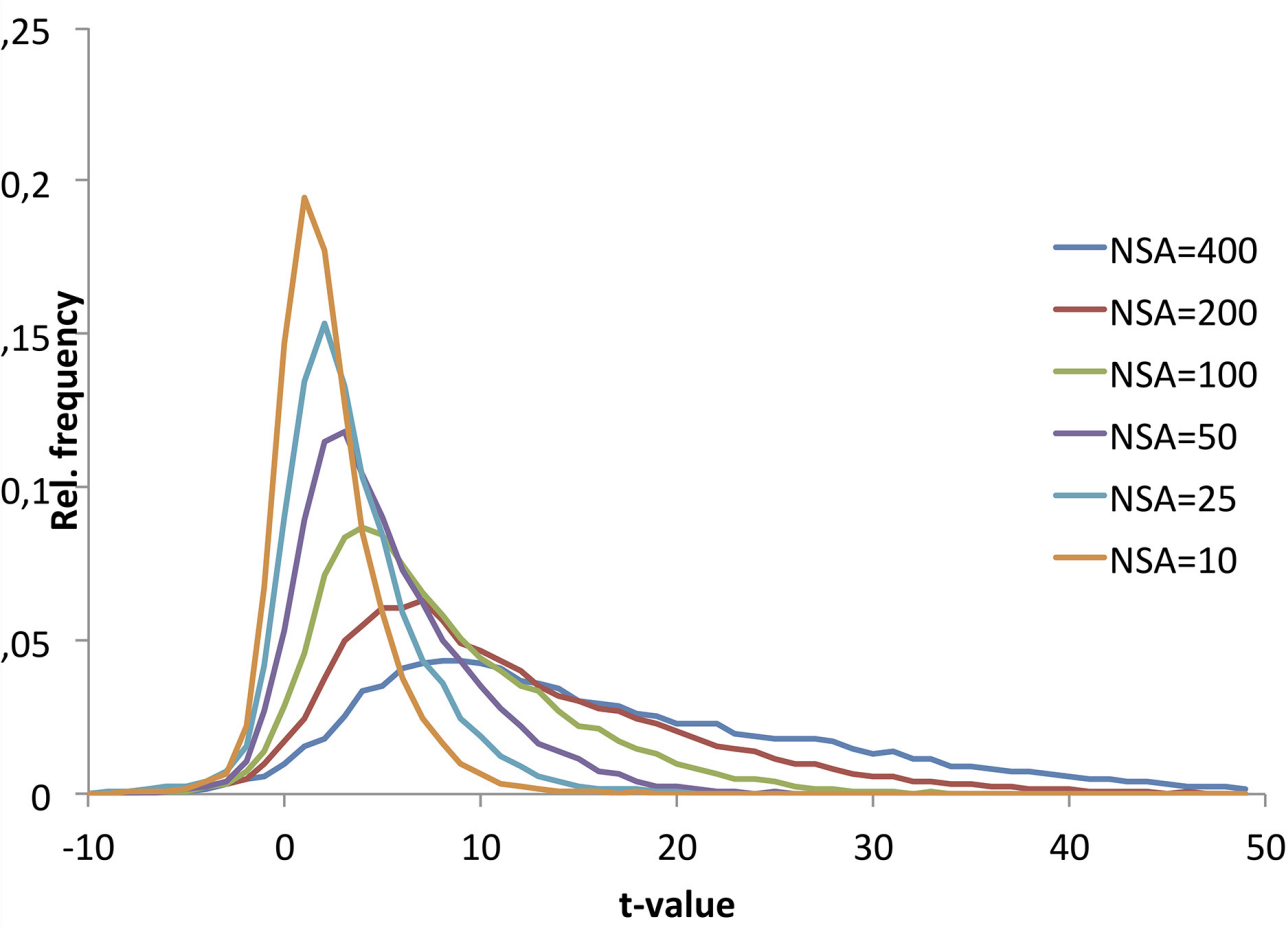
Normalized histograms of distribution of t-values in WM as a function of NSA. Note the normal t-value distribution at low NSA with increasing skewedness of the distributions at higher NSA.

**Fig 9:**
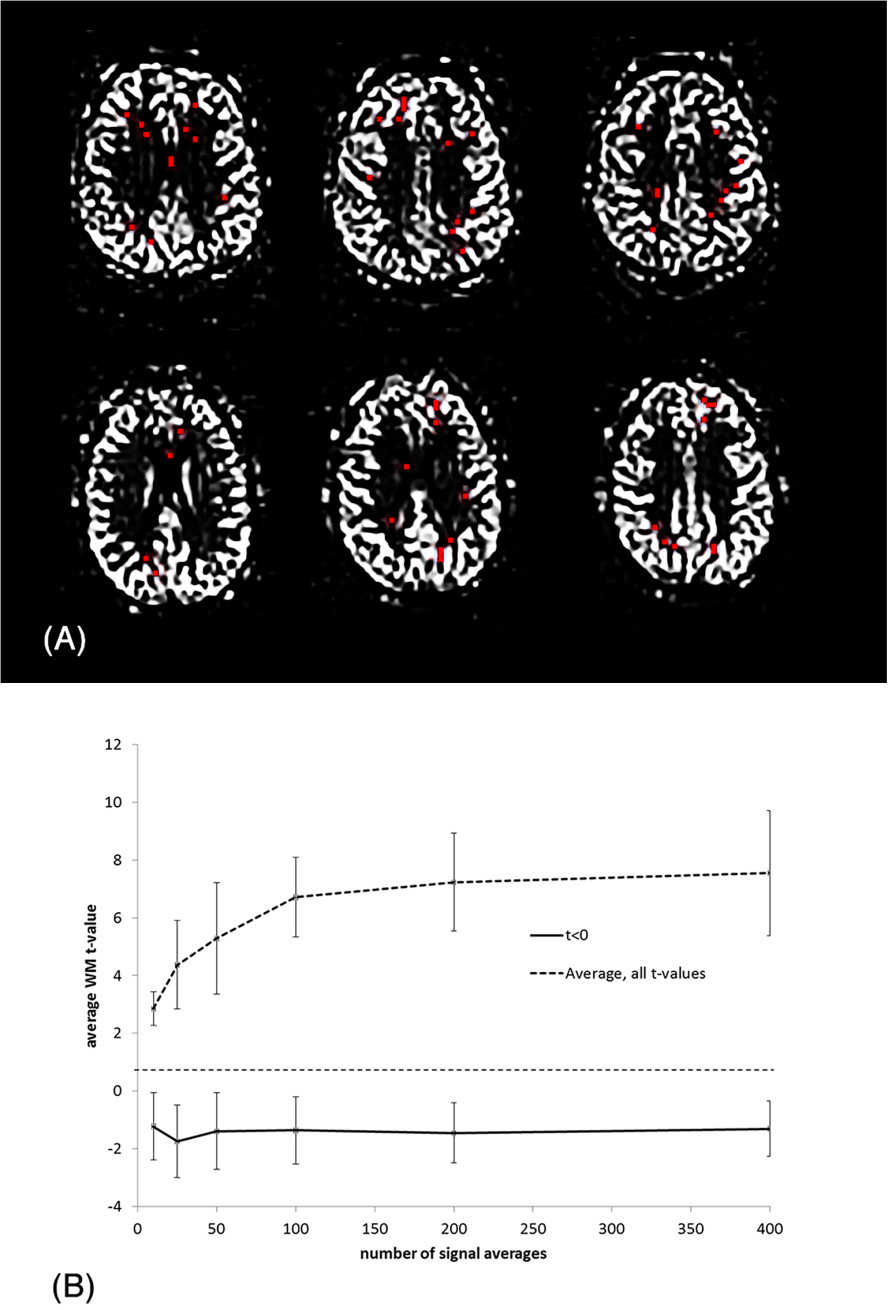
(A) Distribution of ‘non-responding’ voxels (defined as t<0 at all NSA levels) in two representative subjects. No apparent clustering of these voxels is observed. (B) Plots showing average t-value versus NSA for non-responding voxels (t<0) compare to the average for all WM voxels in the same two sample subjects as shown in Fig 9A. Note the lack of NSA-response in the sub-group of voxels shown in red in Fig 9A.

Fig 8 shows the result of the histogram analysis. At low NSA the t-values were normally distributed whereas increasing NSA resulted in an increasingly skewed distribution with an asymmetric tail of increasingly higher t-values but without a corresponding shift in the most negative t-values. The location and distribution of those voxels not responding to increasing NSA (defined as t<0 at all NSA values) are shown in Fig 9A from three slices in two sample subjects. As shown, the voxels appear to be randomly distributed in WM and not clustered to certain WM regions. Fig 9B shows plots of the average t-value progression with increasing NSA for the voxels shown in red in Fig 9A (t<0), and in comparison, the mean t-value progression for all voxels. As shown, for a small fraction of WM voxels the average WM t-value remains negative and is independent of NSA.

**Fig 10:**
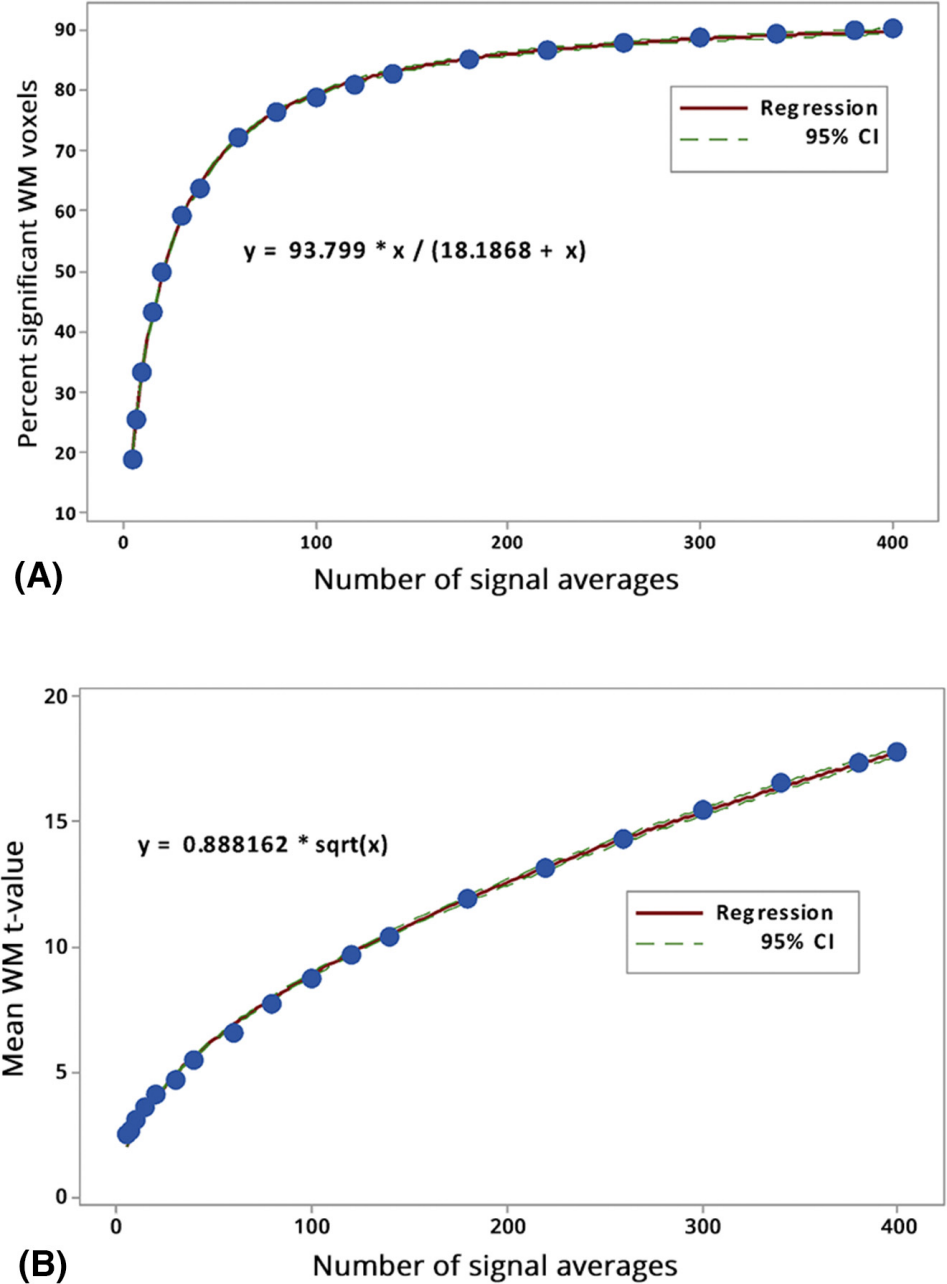
(A) Plot of NSA versus percent significant WM voxels averaged across all subjects (blue dots) and the corresponding curve fit (red line) and the 95% confidence interval for the regression (green lines). (B) Plot of NSA versus mean WM t-value per subject averaged across all subjects (blue dots) and the corresponding curve fit (red line) and the 95% confidence interval for the regression (green lines).

The dependence of average percent significant WM voxels on NSA (Fig 10A) was found to be best described by expression 3. This expression is known to describe so-called Michaelis–Menten kinetics processes [23], giving the rate of enzymatic reactions as a function of substrate concentration (x) and maximum reaction rate (a). Interestingly, the maximum percent of significant WM voxels according to the curve fit was not 100% but about 94%. This further supports the hypothesis that a certain small fraction of WM voxels are not accessible by ASL regardless of NSA. As seen in Fig 10B, mean WM-t-value averaged across all subjects increased with the square root of NSA. This is as expected according to the definition of the t-score:

$$T=u\frac{\surd N}{s.d.}$$

where u = average ASL signal (label-control difference), s.d. = standard deviation of the difference and N is number of observations (= NSA).

## Discussion

This current study is the first to show that it is possible to obtain significant perfusion signal from the vast majority of voxels in the WM by means of a 3T BS PCASL protocol optimized for WM. Obtaining significant perfusion signal from most of the WM voxels comes at the price of very long scan times, as a result of a asymptotic approach to full WM ASL-response with increasing NSA In the most optimal sequence (LD/PLD = 2150 ms/1500 ms) 80% of the voxels were measured to have an ASL-signal significantly larger than zero after five minutes (40% after Bonferroni correction). After this steep initial phase, where a majority of voxels are “filled in”, the remaining population of WM voxels needed extremely long scan times and signal averaging to reach a reasonable significance level, and this signal propagation followed an asymptotic fashion, much in accordance with GM signal propagation, as reported in [9]. The reported percentage is possibly an overestimation, since the entire WM volume was not covered and the included WM sub-volume was relatively homogeneous, potentially leaving out WM regions of poor perfusion properties or with deviant transit time characteristics. The main finding still holds, that the gain from increasing NSA is much higher at low NSA than at high NSA due to the asymptotic response function. As an example, increasing NSA by a factor of 4 from 20 to 80 increased the number of significant WM voxels by about 25% whereas increasing NSA by the same factor from 100 to 400 (a 40 minutes increase in scan time) only further increased the number of significant voxels by about 3%.

The definition of ‘significant ASL signal’ is clearly dependent on the choice of significance level in the statistical analysis as shown in Fig 5. In compliance with previous analysis [9] we used a one-tailed t-test with α = 0.05 for the main analysis, but also tested more stringent α-values, including a full Bonferroni correction for multiple comparisons. The correct choice of significance level is not straightforward and depends, amongst other things, on what is defined as dependent and independent observations. Comparing the NSA response for different α-values suggests that the results are quite similar even for a five-fold reduction in the α-value, whereas a full Bonferroni correction leads to a much larger reduction is significant WM voxels, especially at low NSA. However, Bonferroni correction is probably over-conservative in this context.

The current study was performed as a pilot to a PCASL study of WM pathophysiology of WM lesions in dementia spectrum disorders. The outline of the protocol in this study was based on previous 3T work by van Osch et al [9], which showed that with a PCASL sequence with settings optimal for GM perfusion imaging, significant signal from 70% (not corrected for multiple comparisons) of the WM could be detected at a clinically feasible imaging time of 10 minutes and also claiming, based on a power calculation, that a robust PCASL signal from *all* voxels of the WM would be achievable with around 20 minutes scan time. The same study also showed that the combined prolongation of LD and PLD was deleterious to the amount of WM-voxels showing significant ASL signal. The latter result was more extensively studied by Wu et al [16], who showed in an optimization study that prolonging the LD to 2s provided improved ASL-signal in the WM, whilst PLD prolongation did not, due to excessive label loss from T_1_ relaxation.

Results from our experiments support the claim that a sequence optimized for WM imaging by prolonging the LD performs better. This is in congruence with the study of Wu et al [16], but neither study has found a specific LD that would result in significant signal in *all* WM-voxels. We also reproduce a significant WM signal in “a majority” of voxels within “clinically acceptable” scan times; our somewhat better activation fractions of WM may be explained by the use of a larger voxel size and less parallel imaging acceleration[19] as well as using a very good head immobilization scheme. There was also a better utilization of BS, which is progressively inefficient for later acquired slices when using 2D readout modules, through acquiring fewer slices.

The histogram analysis (Fig 9) reveals an interesting shift in the distribution of WM t-values with increasing NSA. Whereas the upper tail of the distribution is shifted in proportion to increase in NSA, the lower tail does not follow the same trend but is ‘fixed’ in the same low t-value region. This leads to an increasingly skewedness in the t-value distribution with increasing NSA. This trend suggests that a certain small proportion of WM voxels do not respond to an increase in NSA.

This is exemplified in Fig 9 showing the absence of NSA response in those voxels with t-values <0 as extracted from the NSA = 400 data. Further, for increasing NSA, a proportionally smaller portion of WM voxels benefit from further increase in NSA, a hypothesis also supported by the asymptotic shape of the measured increase in average WM t-value with increasing NSA (Fig 10B). The most readily accessible population for detection consists of the most peripheral WM voxels, at the GM/WM interface. Here, PV and PSF effects may cause the signal propagation to be partially driven by GM signal contamination [16,18]; this in contrast to the deepest WM, which will have the longest arrival times and possibly the lowest perfusion.

A major corruptor of data quality in MRI is intra-session head movement, which usually becomes increasingly severe with prolonged scanning. Whilst algorithms can be used to correct for some of this movement, such approaches tend to perform worse with accumulated or large head movement. The presented study had virtually no motion artifact. However, it should be borne in mind that even though this study used an immobilization strategy that prevented head movement during very long scans, the described immobilization procedure is impossible to implement in patient populations, and borderline in healthy subjects, except for in very special experiments, where the volunteer should be comfortable with the scanner environment and trained in endurance of the immobilization device.

Since this study was performed on young, healthy, experienced test subjects, it is to be expected that acquisition and measurements in clinically relevant populations would present lower fractions of significant voxels per unit time and that acquisition times would have to be even longer than what was applied here. Since the majority of WM clinical questions concern detecting hypoperfusion phenomenona, the results of this study represent the “best case scenario” which cannot be generalized for all populations, and suggest that ASL is presently an unsuitable technique at 3T for voxel-wise characterization of WM-perfusion in the deep WM. This might exclude potential clinically interesting studies into e.g. neurodegeneration. Voxel-wise perfusion measurements give detectable signal in more peripheral WM within clinically acceptable scan times, however, some of this signal must be assumed to be of mixed origin due to PV from GM [18] and thus not necessarily representing pure WM perfusion. Prolongation of scan times beyond 10 minutes showed very limited improvement in filling the remaining WM.

Thus; even with optimization of a state-of-the-art labeling module at 3T, a significant fraction of the PCASL signal from WM is so close to the noise level that the method may be unsuitable for voxel-wise assessments of perfusion in WM, and one must rely on averaging of larger areas of WM to make valid inference. A reliable signal comes at the price of extreme acquisition times, whilst the problems of PV effects and intra-session head movement in very long acquisitions must be combatted using additional techniques. The study of deep WM physiology and pathophysiology at a voxel-wise level using ASL at 3T requires further technological advancement, and making strong inference about voxel-wise WM perfusion using present 3T technology should be done with caution.

Finally, studying WM perfusion may benefit from being performed on ultra-high-field scanners, which gives access to higher SNR and longer T_1_ blood relaxation times. A recently published 7T study reported that a reasonable signal from 75% of the WM could be measured in an 11 minute ASL scan at 3×3×5 mm^3^ resolution, using a pulsed ASL approach [13], whereas PCASL approaches at 7T suffer from B1 and B0-issues as well as high specific absorption rate (SAR) that leads to a highly inefficient increase in TR [24]. When comparing this with our results showing significant signal in 87% after the same scan time with 3.75×3.75×5 mm^3^ voxels, it is clear that higher magnetic field strength does not guarantee sufficient detection power to measure deep WM ASL-signal.

## Conclusion

Complete voxel-wise measurements of CBF using a state-of-the-art PCASL approach at 3T may only be possible using extreme scan times that are incompatible with clinical practice. For clinical assessments, averaged ROI-based flow quantification may well be feasible and valid.
